# Salivary cell-free DNA methylation analysis for oncological monitoring of surgical resection of oral squamous cell carcinoma

**DOI:** 10.3389/froh.2025.1614371

**Published:** 2025-06-11

**Authors:** Zuzana Saidak, Marie Milly, Christophe Louandre, Emilien Colin, Pia-Manuela Rusu, Agnes Paasche, Stephanie Dakpe, Sylvie Testelin, Antoine Galmiche

**Affiliations:** ^1^ UR7516, CHIMERE, Université de Picardie Jules Verne, Amiens, France; ^2^ Service de Biochimie, Centre de Biologie Humaine, CHU Amiens, Amiens, France; ^3^ Institut Faire Faces, Amiens, France; ^4^ Service de Chirurgie Maxillo-faciale, CHU Amiens, Amiens, France

**Keywords:** oral squamous cell carcinoma, surgical resection, saliva, cell-free DNA methylation, biomarkers

## Abstract

**Objective:**

Non-invasive analysis of tumor DNA in biological fluids offers promising perspectives for the oncological monitoring of cancer patients. Cancer-specific DNA methylation marks are detectable in the saliva of Oral Squamous Cell Carcinoma (OSCC) patients. We set up a salivary liquid biopsy approach for the oncological monitoring of OSCC referred for surgical resection.

**Material and methods:**

We analysed DNA methylation in TCGA-OSCC to identify genes with high methylation levels in tumor vs. matched non-tumor tissue. Cell-free DNA (cfDNA) methylation levels of selected genes were analysed in the saliva of OSCC patients (*n* = 30) before/after complete surgical resection by High Resolution Melting (HRM) analysis, and compared to non-cancer controls.

**Results:**

We identified five genes with higher DNA methylation levels in OSCC compared to matching non-tumor tissue that were analysable by HRM, and were independent of tumor stage, etiology or age. In 70% of OSCC, at least one of the five cfDNA methylation marks was detectable before surgery. Complete surgical resection led to a significant disappearance of salivary cfDNA methylation marks. In 52% of patients, we noted the persistence of at least one mark, shown to be related to close/positive surgical margin status. In one patient resected with R0 margin, the persistence of *ASCL1* methylation preceded tumor recurrence by 4 months.

**Conclusion:**

Salivary cfDNA methylation analysis offers a minimally invasive method to monitor the effectiveness of surgical resection of OSCC. Future studies with a larger cohort and longer follow-up are required to validate its use in this context.

## 1 Introduction

Oral squamous cell carcinoma (OSCC) is a relatively common malignancy arising from the epithelial lining of the oral cavity, usually in a context of chronic exposure to tobacco and alcohol (1–4). In most cases, surgical resection with curative intent represents the first line of treatment. Despite advances in therapy, OSCC still poses a significant clinical challenge. The high frequency of postsurgical recurrence accounts for the 5-year overall survival (OS) rate of around 60%. The follow-up of OSCC currently relies on clinical examination and tumor imaging. Given the complex nature of surgical procedures, the existence of multiple perioperative protocols and the intrinsic heterogeneity of clinical situations, predicting postsurgical recurrence of OSCC, remains difficult (5). Therefore, an objective, biology-based method for evaluating the completeness and efficacy of surgical resection in OSCC remains an unmet clinical need (6).

The identification of sensitive biomarkers of malignancy and their application for non-invasive cancer detection through liquid biopsies—i.e., the analysis of cancer-derived material in blood or other biological fluids—represents a promising strategy for improving cancer management (7–9). Some of the most interesting applications of liquid biopsies include improving the diagnosis of cancer, identifying Minimal Residual Disease (MRD) and optimizing the oncological follow-up after cancer therapy (7, 8). DNA methylation is a well-characterized epigenetic mechanism that plays a central role in regulating gene expression in eukaryotic cells (10). Aberrant methylation patterns are closely associated with the malignant phenotype and are frequently observed in tumor-derived DNA. Importantly, these methylation marks can be detected in cell-free DNA (cfDNA) released by tumor cells into biological fluids, such as blood or saliva. The detection of methylation marks in cfDNA underpins many current liquid biopsy approaches. Analysis of tumor-specific cfDNA methylation marks offers valuable diagnostic insights and has shown promise in the early detection of cancer. In oral squamous cell carcinoma (OSCC), non-invasive detection of specific methylation signatures in cfDNA—particularly from blood or saliva—may improve the identification of malignancy in the setting of oral potentially malignant lesions (11). The diagnostic performance of these assays varies depending on the genes analyzed (11). Beyond diagnosis, cfDNA-based liquid biopsies also hold significant potential for monitoring cancer patients during treatment and follow-up. The detection of tumor-specific cfDNA methylation marks could reflect MRD and enable the early identification of tumor recurrence (7, 8). In a recent study, analysis of the plasma cfDNA methylome in OSCC patients revealed the presence of tumor-derived methylation marks in blood samples collected prior to surgical resection (12). These findings support the feasibility of using cfDNA methylation profiling for perioperative disease monitoring in OSCC (12). While the study highlights the diagnostic potential of cfDNA methylation analysis, its design and the gene panel employed may not be optimal for postoperative oncological surveillance. Notably, histologically normal tissues adjacent to the tumor, i.e., surgical margins, can exhibit epigenetic alterations similar to those found in the tumor itself (6, 13). In the surgical context, for the monitoring and follow-up of OSCC resection, a better strategy would involve analyzing the genes with the highest differential DNA methylation between tumors vs. surgical margins. Furthermore, the anatomical accessibility of OSCC offers a unique opportunity for salivary cfDNA analysis. Compared to plasma-based assays, saliva sampling provides proximity to the tumor site and may enable more sensitive and specific detection of tumor-derived methylation signatures, offering optimal conditions for “local tumor sampling” (8).

In this study, we developed a salivary liquid biopsy approach for monitoring patients with OSCC undergoing surgical resection. Using data from The Cancer Genome Atlas (TCGA) OSCC cohort (14), we identified genes exhibiting the highest differential methylation between tumor and matched non-tumor tissues. From this analysis, five candidate genes were selected for evaluation in salivary samples collected from OSCC patients prior to and after surgery. We then assessed whether the persistence of their methylation marks postoperatively correlated with the oncological outcome of surgical resection.

## 2 Materials and methods

### 2.1 DNA methylation analysis in OSCC and matching non-tumor samples

We used the Head and Neck Squamous Cell Carcinoma (HNSC) cohort from TCGA to obtain gene methylation data (Beta-values) for *n* = 321 OSCC (Illumina Infinium HumanMethylation450 BeadChip assay, HM450) (15, 16). Paired methylation data for tumor/non-tumor samples were available for 32 patients, retrieved through Firebrowse, http://firebrowse.org/ in January 2023. Clinical information was retrieved through cBioportal (https://www.cbioportal.org/) in January 2023 and is summarized in Table 1.

**Table 1:** Clinical description of the cohorts analyzed.

	TCGA-OSCC (*n* = 32)	EPSACO patients (*n* = 30)	EPSACO controls (*n* = 10)
Age (years) Median [range]	61 [26–87]	65 [35–80]	56 [37–62]
Sex	Male *n* = 21	Male *n* = 19	Male *n* = 6
	Female *n* = 11	Female *n* = 11	Female *n* = 4
Tobacco^a^	Yes *n* = 23	Yes *n* = 25	Yes *n* = 8
	No *n* = 9	No *n* = 2	No *n* = 2
		NA *n* = 3	
Alcohol^b^	Yes *n* = 22	Yes *n* = 16	Yes *n* = 10
	No *n* = 10	No *n* = 3	No *n* = 0
		NA *n* = 11	
Primary location	Tongue *n* = 22	Tongue *n* = 13	
	Oral cavity *n* = 5	Oral cavity *n* = 9	
	Floor of mouth *n* = 4	Floor of mouth *n* = 6	
	Palate *n* = 1	Palate *n* = 2	
Stage^c^	Stage I/II *n* = 9	Stage I/II *n* = 8	
	Stage III/IV *n* = 23	Stage III/IV *n* = 19	
		NA *n* = 3	
Surgical margin^d^	Negative *n* = 26	Negative *n* = 17	
	Positive *n* = 2	Positive *n* = 2	
	Close *n* = 1	Close *n* = 10	
	NA *n* = 3	NA *n* = 1	

NA, not available.

^a^ Tobacco history encompasses both current and former use.

^b^ Alcohol history includes any level of prior consumption, including occasional use.

^c^ TCGA-OSCC staging is based on AJCC 7th edition, EPSACO staging is based on the 8th edition.

^d^ Close margin defined as <5 mm.

### 2.2 Saliva sample collection from surgical OSCC patients

To collect salivary samples before and after surgery, we conducted a prospective, single-center study at Amiens University Hospital (France) between June 2022 and March 2024, involving patients with OSCC scheduled for surgical resection. The EPSACO study received approval from the Comité de Protection des Personnes Sud-Est VI (CPP 2022-A00723-40) and is declared on ClinicalTrials.gov (NCT05791149). Thirty patients with histologically proven OSCC referred to the department of Maxillofacial Surgery for surgical resection were recruited (clinical information summarized in Table 1). Informed consent was obtained from all participants. Salivary samples were collected immediately before the surgical resection of OSCC and 4 weeks after surgery. Pathology reports were used to retrieve the surgical margin status information. The median follow-up of included patients was 24 months (range 11–32 months). We also collected saliva samples from 10 controls, i.e., non-cancer patients matched for age, sex and tobacco/alcohol intake.

### 2.3 DNA extraction and methylation analysis from saliva samples

Saliva samples were collected using SpeciMAX™ Saliva Collection Kit (Thermo Fisher). Samples were transferred to the laboratory and processed immediately after collection. They were spun for 5 min at 500 g to remove any cells, keeping only the supernatant, then frozen at −80°C ahead of analysis. Salivary cfDNA was extracted using MagMAX™ Cell-Free DNA Isolation Kit (Thermo Fisher). We assessed DNA quality and concentrations with NanoDrop microvolume spectrophotometer, after DNA purification and after bisulfite conversion. Bisulfite conversion was performed on all DNA samples and controls using the EpiJET Bisulfite Conversion Kit (Thermo Fisher). Fully methylated (CpG methylated Human Genomic DNA, Thermo Fisher) and unmethylated control DNA (CpGenome™ Universal Unmethylated DNA Set, Merck) were used as controls for bisulfite conversion. High Resolution Melting (HRM) analysis was used to examine DNA methylation levels at specific loci (17). Briefly, a melting analysis was performed on short PCR products including the CpG islands near the promoter of the genes of interest. The melting profiles from OSCC samples were compared to a methylation range prepared using fully methylated (100%) and non-methylated (0%) reference samples (MeltDoctor™ HRM Master Mix, Thermo Fisher). Primers were designed using the MethPrimer tool (https://www.urogene.org/cgi-bin/methprimer/methprimer.cgi), with the option of bisulfite sequencing (Supplementary Table S1). HRM methylation analysis was performed on a QuantStudio 7 Real-Time PCR System (Thermo Fisher Scientific) using the Methylation Analysis Module (HRM Plugin) for digital analysis of DNA melting profiles. The DNA methylation of each gene (beta-value) was calculated to be between 0 (non-methylated) and 1 (fully methylated). The detection limit of this assay was determined to be at 1%. A representative example of HRM analysis for *ASCL1* is shown in Supplementary Figure S1.

### 2.4 Statistical analysis

All statistical analyses were performed on GraphPad prism software and R (https://cran.r-project.org) (packages ggplot, dplyr, ggthemes, pROC, gplots). For the description of population characteristics, quantitative variables are described using the median (minimum - maximum). Qualitative variables are described by their frequency. Chi-squared analysis was applied for categorical data, Wilcoxon signed-rank test or Student's t-test for numeric data. Association of methylation mark to disease status was assessed with the Wald test (Relative Risk). Risk alpha was set to 5%. False discovery rate (FDR) correction was applied as indicated. Classifier performance was evaluated with the Receiver Operating Characteristic (ROC) analysis, calculating the AUC (Area Under the Curve). ROC performance parameters were calculated using the R package pROC.

## 3 Results

### 3.1 Comparisons of DNA methylation marks between OSCC and matched non-tumor margins

**Figure 1:**
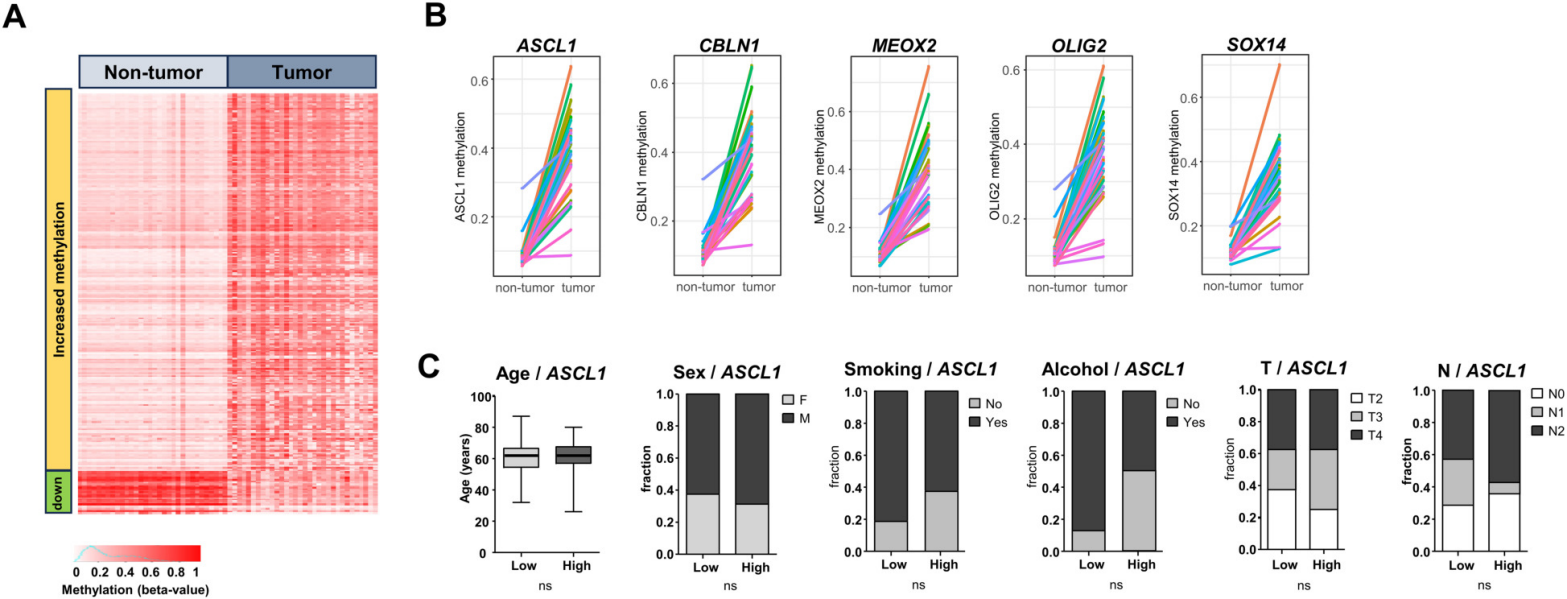
Comparison of DNA methylation in TCGA-OSCC vs. matched non-tumor samples. **(A)** Heatmap showing all significantly differentially methylated genes in matched tumor OSCC/non-tumor samples from TCGA. Red indicates fully methylated samples (beta value 1), white indicates non-methylated samples (beta value 0). **(B)** Five of the top genes identified to be significantly more methylated in tumor samples vs. matched non-tumor samples, that were kept for the present analysis (*ASCL1*, *CBLN1*, *MEOX2*, *OLIG2*, *SOX14*). **(C)** Lack of link between *ASCL1* methylation levels and age, sex, smoking history, alcohol history and T and N categories. Tumors were stratified according to the median *ASCL1* DNA methylation to define low/high groups. Student's t-test or Chi2, *p* < 0.05 used as threshold for significance.

A pan-genomic comparison of DNA methylation levels (HM450) in paired tumor/non-tumor samples from TCGA-OSCC (*n* = 32 matched pairs) led to the identification of 248 genes that were significantly more methylated in OSCC compared to non-tumor samples (>2 fold difference, *p* < 0.05 in Wilcoxon signed-rank test with FDR correction) (Figure 1A; Supplementary Table S2). This analysis also identified 28 genes that were significantly less methylated in OSCC (Figure 1A). From the list of 248 genes that were more methylated, we selected the top 10 protein coding genes with the highest differential methylation and the most significant *p*-values, and we tested their suitability for HRM analysis with the MethPrimer tool. Five genes were found to be suitable for HRM analysis: *ASCL1, CBLN1, MEOX2, OLIG2* and *SOX14*. Line plots showing the DNA methylation levels of these genes in OSCC vs. matching non-tumor samples are shown in Figure 1B. Given the variety of clinical situations encountered with OSCC, we examined the possibility that DNA methylation at the selected loci might be related to clinical features. As shown in Figure 1C, the representative analysis performed for *ASCL1* suggested that its associated DNA methylation marks were equally present in all OSCC, regardless of staging, age, sex or exposure to tobacco and alcohol. The same analysis performed for each of the five genes gave comparable results (Supplementary Table S3).

### 3.2 Salivary DNA methylation profile of surgical OSCC patients

**Figure 2:**
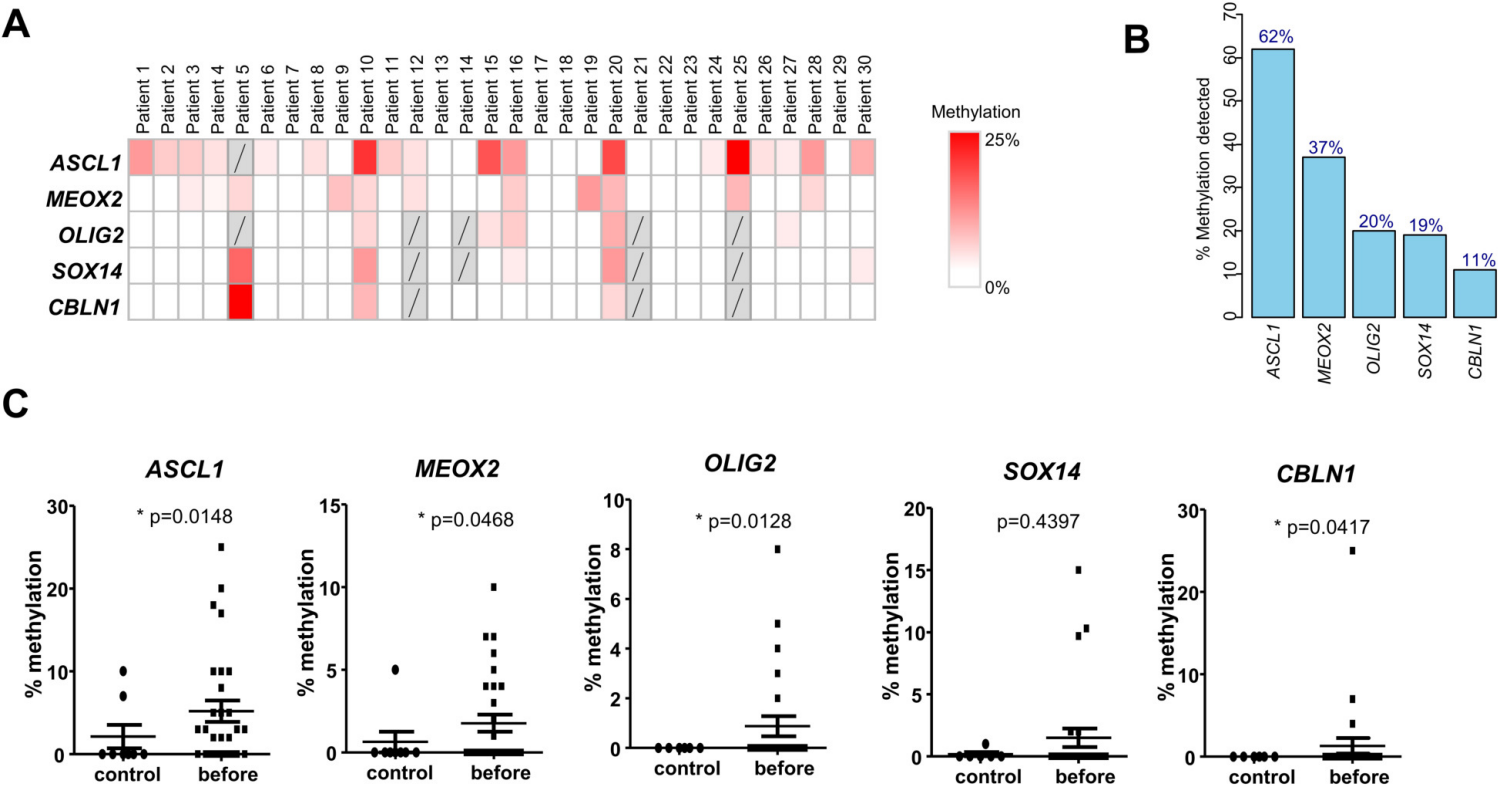
DNA methylation in saliva of OSCC patients before surgical resection. **(A)** Methylation levels of the selected five genes in saliva samples from 30 prospectively recruited OSCC patients before surgical resection. **(B)** Percentage of patients with methylation of the five genes detected before surgery, in descending order. **(C)** Comparison of methylation levels of the five genes in saliva of OSCC patients before surgery vs. age/sex-matched healthy controls (Wald test).

In order to examine the DNA methylation of the selected five genes in OSCC, we prospectively collected and analysed the saliva from *n* = 30 OSCC before surgical resection. Using HRM analysis on the five previously identified genes, we noted that each DNA methylation mark was detectable at different intensities and variable frequencies (Figure 2A). The average % of methylation in positive samples were: *ASCL1* 8.4%, *MEOX2* 4.8%, *CBLN1* 12%, *OLIG2* 4.4%, *SOX14* 7.8%, well above the technical threshold of the HRM technique (Figure 2A). While DNA methylation marks on *ACSL1* and *MEOX2* genes were detectable in 62% and 37% of OSCC, respectively, methylation marks on *OLIG2*, *SOX14* and *CBLN1* were found in <20% of OSCC, as shown in Figure 2B where the genes are ranked by decreasing frequency of cfDNA methylation (Figure 2B). No salivary cfDNA methylation marks were detected in 9 out of the 30 pre-operative OSCC, i.e., 30%. When we examined the patients with at least one positive cfDNA methylation mark, a significant overlap of the different cfDNA methylation marks was evident. A combined DNA methylation analysis of *ASCL1, MEOX2* and *OLIG2* was sufficient to detect 70% of pre-operative OSCC (21 out of 30) (Figure 2A). A direct comparison between OSCC and age/sex and tobacco/alcohol matched non-cancer controls further suggested the clinical interest of these cfDNA methylation marks (Figure 2C). The methylation levels of *ASCL1*, *MEOX2*, *OLIG2* and *CBLN1* were found to be significantly higher in OSCC than non-cancer controls (*p* = 0.0148, *p* = 0.0468, *p* = 0.0128 and *p* = 0.0417, respectively), while *SOX14* did not reach statistical significance. The data suggested that salivary DNA methylation analysis is applicable to surgical OSCC.

### 3.3 Detection of the salivary cfDNA methylation profile before and after surgery

**Figure 3:**
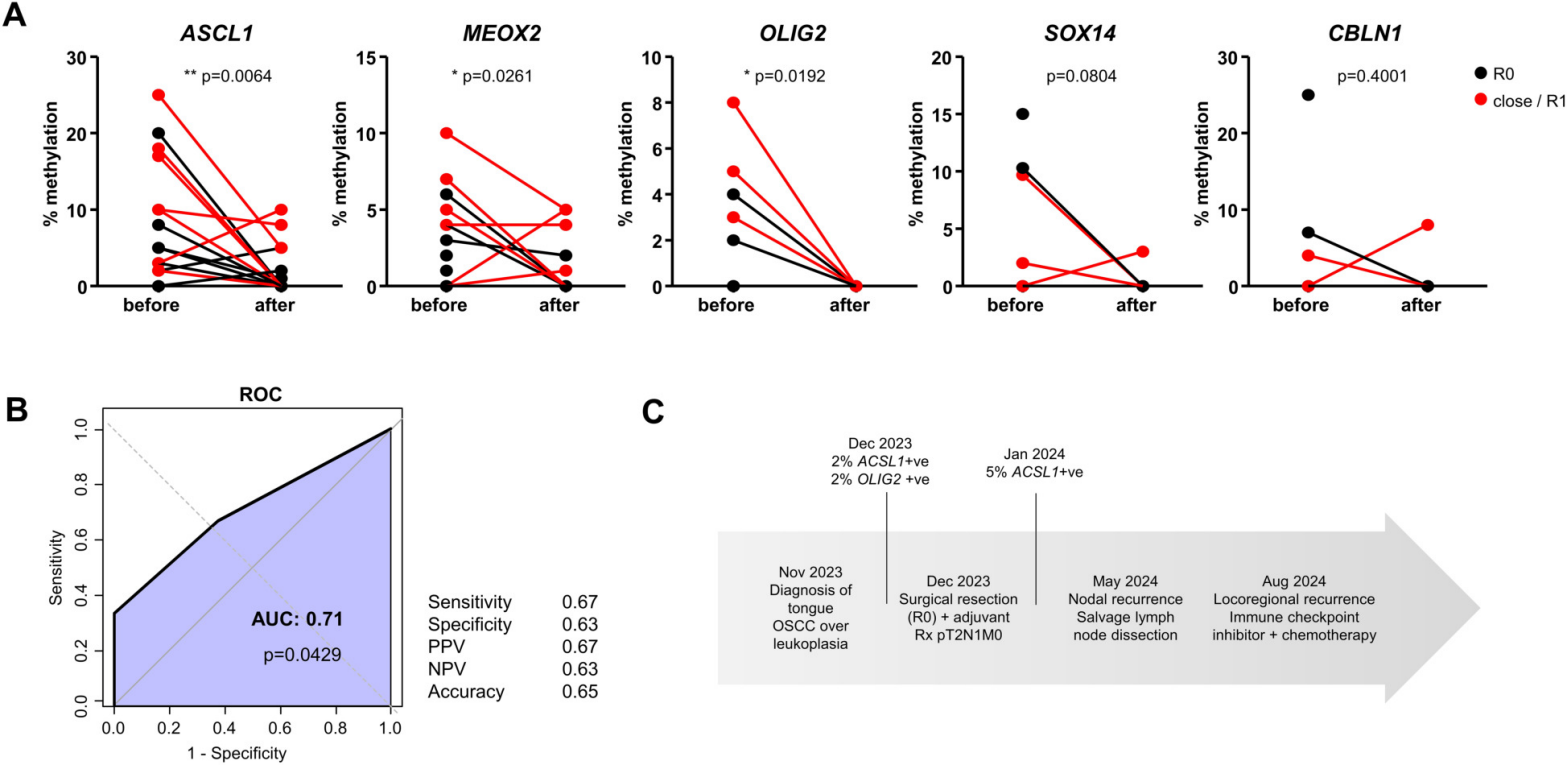
Evolution of DNA methylation marks after surgery. **(A)** Methylation levels of the five genes before surgical resection and after surgical resection (paired Student's t-test). Patients with negative surgical margins (R0) are shown in black; patients with close/positive surgical margins (R1) are shown in red. **(B)** ROC analysis of the persistence of methylation detected after surgical resection and the detection of close/positive surgical margins. Performance parameters were calculated using 1 positive methylation mark after resection as cutoff (PPV = positive predictive value, NPV = negative predictive value) in *n* = 17 patients. **(C)** Case study of a patient with persistent methylation after resection, categorized as R0 that relapsed shortly after.

Out of the 21 pre-surgical salivary samples with at least one DNA methylation mark, a second sample was available for 17 patients after surgery (4 weeks after resection). We compared the evolution of cfDNA methylation rates for the five selected genes before and after resection (paired Student's t test). The levels of cfDNA methylation decreased for all 5 genes after surgery, reaching statistical significance for *ASCL1*, *MEOX2* and *OLIG2*, suggesting their reflection of the tumor mass (Figure 3A). Among the 17 patients for which matching before/after samples were available, a complete eradication of all methylation marks was noted in 47% of patients (8/17). Next, we aimed to compare the results of DNA methylation analysis with the outcome of surgical resection, defined as the existence of negative surgical margins (R0) vs. close/positive margin (defined as R1 or tumor margins <5 mm upon pathological examination of the resected OSCC). A ROC analysis shown in Figure 3B indicated a significant association between a close/positive margin and the persistence of ≥1 DNA methylation mark 4 weeks after surgery (AUC = 0.71, *p* = 0.0429). In our population, the persistence of at least one positive cfDNA methylation mark reflected a close/positive margin with sensitivity of 0.67, specificity of 0.63, Positive Predictive Value (PPV) of 0.67, Negative Predictive Value (NPV) of 0.63 and Accuracy of 0.65 (Figure 3B). We then explored the performance of individual genes and gene combinations (Supplementary Table S4). In individual gene or combined ROC analyses we show that none of the genes performed well on its own. Three different combinations of genes performed the best with an AUC > 0.71 [and *p* < 0.05 (Supplementary Table S4). All combinations included the genes *MEOX2* and *CBLN1*, but none included *OLIG2*. A thorough disease-free survival analysis could not be performed due to incomplete data availability (a high % of lost to follow-up patients, and a limited duration of follow-up). Interestingly however, in one patient resected with R0 margin, the persistence of *ASCL1* methylation preceded tumor recurrence by 4 months (Figure 3C).

## 4 Discussion

Surgical resection with curative intent represents the first line of treatment for most OSCC. The detection of MRD after curative treatment could help identify cancer patients at higher risk of recurrence, potentially suggesting the need for close clinical and radiological monitoring and possibly more intensive adjuvant treatment. It could also provide a proxy for classical survival analysis for research protocols and studies examining the operative and perioperative procedures. In the present study, we analysed tumor-specific cfDNA methylation marks in the saliva of OSCC, a convenient matrix that is easily collectable and previously reported to allow for non-invasive analyses of nucleic acids shed by OSCC (11). We based our study on the top genes identified in a survey of genes with the highest differential DNA methylation status between OSCC and non-tumor tissue, five of which could be analysed by HRM. We verified that these DNA methylation marks were broadly analysable in the multiple clinical situations found in OSCC. Three of these DNA methylation marks decreased or disappeared after curative resection of OSCC. We found a significant association between a close/positive margin and the persistence of ≥1 DNA methylation mark 4 weeks after surgery, a finding that validated the principle of our analysis. In one patient presenting with an apparently successful R0 margin, the persistence of *ASCL1* methylation preceded tumor recurrence by 4 months. The technical approach applied here represents a novel, tumor-agnostic, easily implementable and cost-effective strategy for the objective oncological monitoring of surgical resection of OSCC.

A clear weakness of our study is the limited recruitment of patients, with a short follow-up and high % of lost to follow-up patients, preventing us from testing whether the persistence of tumor-specific salivary cfDNA methylation marks correlates with post-surgical recurrence. Well-conducted studies with a larger recruitment and a better follow-up will be required in order to validate the principle of cfDNA methylation analysis. Our study also highlights a potential weakness of the technical approach, i.e., its incomplete sensitivity. This is an important problem that was recently discussed elsewhere (8). Compared to PCR-based detection of viral genotypes or the detection of missense DNA mutations or DNA methylation analyses using droplet digital PCR (18), the classical HRM analysis is likely intrinsically less sensitive. While the classical HRM approach is probably not ideal for the sensitive diagnosis of MRD, technical solutions, including its transposition to digital PCR platforms, exist, as reported elsewhere (19).

Compared to previous literature addressing the interest of DNA methylation analyses for the diagnosis of OSCC, the DNA methylation marks that we selected here were customized for the surgical context, based on the comparison of OSCC with non-tumor tissue from TCGA. This strategy identified genes with little or no overlap with those reported in previous works (11). The five genes that we identified here as highly differentially methylated in OSCC vs. non-tumor tissue, i.e., *ASCL1*, *CBLN1*, *MEOX2*, *OLIG2*, and *SOX14*, are primarily involved in developmental and neuronal processes (20–24). *ASCL1* is a transcription factor essential for neuroendocrine differentiation and is upregulated in several neuroendocrine tumors, including small cell lung cancer, where it promotes cell proliferation and survival (20). *CBLN1*, typically associated with synaptic function, has limited data linking it to cancer, though recent studies suggest it may influence cell signaling in specific malignancies (21). *MEOX2*, a homeobox gene, has been implicated in promoting angiogenesis and tumor progression in glioblastoma and lung cancer through the regulation of the cell cycle and vascular factors (22). *OLIG2*, another neural transcription factor, plays a critical role in glioma development by maintaining cancer stem cell populations and supporting resistance to therapy (23). *SOX14* has been shown to influence tumor cell differentiation and epithelial-mesenchymal transition in some cancers (24). While these studies suggest their potential contributions to carcinogenesis in specific tumor types, there is at this stage no evidence linking their expression or DNA methylation to oral carcinogenesis. Our preliminary analysis suggests that none of the genes that we studied performs sufficiently well on its own. Expanding the cfDNA methylation analysis to include a broader panel of genes may enhance test sensitivity, suggesting potential avenues for improving salivary cfDNA methylation assays.

Besides the molecular diagnosis of MRD, liquid biopsies hold interesting perspectives for the objective assessment of surgical procedures. In our experience, a significant decrease in salivary tumor-specific cfDNA marks was noted, as well as a statistical correlation with the surgical margin status. This strongly suggests the interest of salivary cfDNA analysis as a valid strategy to achieve a rapid objective oncological evaluation of OSCC resections. Importantly, an increasing number of technological developments are becoming available to guide OSCC resection, based for example on fluorescence or mass spectrometry (25, 26). New medical perioperative protocols are also being introduced, such as neoadjuvant treatments based on immune checkpoint inhibitors (27). These new developments will likely change the conditions in which surgical resections are performed, and might also have an important oncological impact for operated cancer patients. However, their oncological evaluation is currently lagging behind since it is dependent on the completion of large clinical studies with a long follow-up. We argue that our liquid biopsy approach could allow a technically simple, post-operative objective assessment of OSCC resections.

## Data Availability

The raw data supporting the conclusions of this article will be made available by the authors, without undue reservation.
