# Supplementary material for: Salivary cell-free DNA methylation analysis for oncological monitoring of surgical resection of oral squamous cell carcinoma
Source: Front Oral Health. 2025 Jun 11;6:1614371. doi: 10.3389/froh.2025.1614371 (PMC12187844; doi:10.3389/froh.2025.1614371)
Supplement: Supplementary file 1 [file Datasheet1.docx]

Supplementary Material

## Supplementary Figures


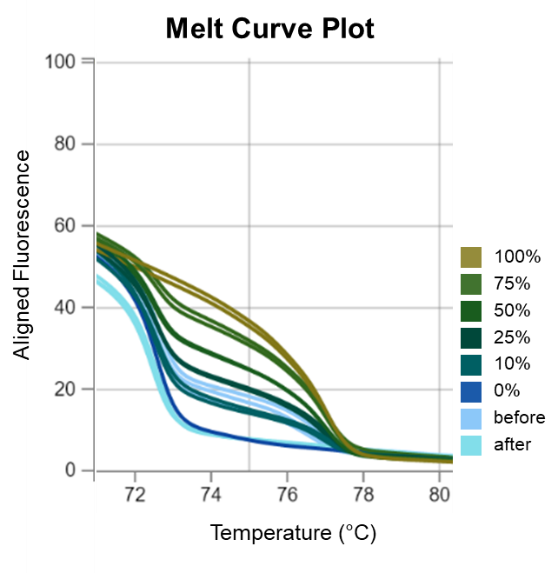


**Supplementary Figure 1.** An example of High Resolution Melting analysis, showing a melt curve plot for Patient #15 (before/after surgery) in comparison to a range of DNA methylation levels for the gene *ASCL1*.

## Supplementary Tables

## Supplementary Table 1: Primer sequences used in this study.

**Gene Forward primer Reverse primer Ta (°C)**

***ASCL1*** ATTTAAGTTTTTTTTGTGTTTTTTT CCATCTTAACAAAACTTTCCATAC 60

***CBNL1*** GGTGTTATTATAGGGGTAAAATTAGG AACCCAACTACCAAAACACAATAAA 60

***MEOX2*** TTTTTTAGTTTGGGTTTTAGTATTT AAAAACAACACATTCCCATCTTC 60

***OLIG2*** TTTTTTTTGGGGTTATGGATT AAAACCACTACCTCCTAACTTATCC 60

***SOX14*** AGGAGTTTTTTGTTTTGGTTAAAAA CCCCACAAAATTCAACTAATCATAC 60

Ta: Annealing temperature

## Supplementary Table 2: List of the 248 significantly differentially methylated genes (fold difference >2) in tumor samples compared to matching non-tumor samples in TCGA-OSCC

**Gene Non-tumor (median) Tumor (median) Fold increase Paired Wilcoxon (FDR)**

*ASCL1* 0.087 0.386 4.44 0.00000937

*WIT1* 0.11 0.476 4.31 0.00000937

*CBLN1* 0.101 0.429 4.25 0.00000937

*MEOX2* 0.098 0.4 4.09 0.00000937

*MIR124-1* 0.085 0.322 3.78 0.00000937

*POU4F2* 0.131 0.481 3.67 0.00000937

*GSX1* 0.105 0.386 3.66 0.00000937

*FOXB2* 0.12 0.43 3.58 0.00000937

*OLIG2* 0.106 0.376 3.54 0.00000937

*MIR196A1* 0.162 0.569 3.51 0.00000937

*MIR124-2* 0.155 0.522 3.37 0.00000937

*NKX2-4* 0.126 0.424 3.36 0.00000937

*NKX2-6* 0.136 0.446 3.27 0.00000937

*SOX14* 0.113 0.366 3.26 0.00000937

*NEUROD1* 0.131 0.424 3.24 0.00000937

*ADCYAP1* 0.136 0.437 3.22 0.00000937

*SLC32A1* 0.113 0.36 3.19 0.00000937

*INA* 0.135 0.427 3.17 0.00000937

*LRRTM1* 0.137 0.429 3.12 0.00000937

*SOX17* 0.171 0.533 3.12 0.00000937

*PCDH8* 0.148 0.448 3.04 0.00000937

*PCDH10* 0.121 0.367 3.02 0.00000937

*HOXB4* 0.175 0.518 2.97 0.00000937

*FLJ43390* 0.125 0.37 2.95 0.00000937

*SLITRK1* 0.141 0.414 2.94 0.00000937

*TLX3* 0.149 0.436 2.93 0.00000937

*BARHL2* 0.165 0.478 2.9 0.00000937

*SLC18A3* 0.142 0.411 2.89 0.00000937

*CR1* 0.145 0.417 2.88 0.00000937

*C14orf39* 0.149 0.426 2.86 0.00000937

*CYP26A1* 0.108 0.306 2.84 0.00000937

*ZNF154* 0.226 0.632 2.8 0.00000937

*HTR1A* 0.146 0.406 2.78 0.00000937

*FEZF2* 0.132 0.364 2.76 0.00000937

*RXFP3* 0.155 0.425 2.74 0.00000937

*LRFN5* 0.139 0.381 2.74 0.00000937

*FEZF1* 0.126 0.335 2.66 0.00000937

*NETO1* 0.199 0.517 2.6 0.00000937

*MIR663* 0.233 0.603 2.59 0.00000937

*SFTA3* 0.161 0.414 2.57 0.00000937

*HOXD13* 0.155 0.397 2.57 0.00000937

*NPY* 0.193 0.493 2.55 0.00000937

*C1QL2* 0.139 0.354 2.55 0.00000937

*HTR1B* 0.157 0.399 2.55 0.00000937

*HOXD8* 0.142 0.36 2.55 0.00000937

*HAND2* 0.172 0.435 2.54 0.00000937

*ZNF542* 0.199 0.505 2.54 0.00000937

*PRAC* 0.15 0.379 2.53 0.00000937

*PDX1* 0.132 0.332 2.51 0.00000937

*ZNF132* 0.209 0.525 2.51 0.00000937

*GAD2* 0.168 0.42 2.5 0.00000937

*ALX1* 0.159 0.394 2.48 0.00000937

*NKX2-1*  0.104 0.256 2.45 0.00000937

*VSTM2B* 0.168 0.406 2.42 0.00000937

psiTPTE22 0.204 0.49 2.41 0.00000937

*HOXA7* 0.185 0.442 2.39 0.00000937

*POU4F3* 0.136 0.323 2.38 0.00000937

*VAX1* 0.198 0.471 2.38 0.00000937

*NMBR* 0.175 0.417 2.38 0.00000937

*DKFZP434H168* 0.143 0.341 2.38 0.00000937

*FERD3L* 0.156 0.371 2.37 0.00000937

*RAX* 0.141 0.332 2.35 0.00000937

*LBX1* 0.102 0.238 2.34 0.00000937

*C17orf93* 0.162 0.378 2.33 0.00000937

*FOXG1* 0.22 0.51 2.31 0.00000937

*GSC* 0.166 0.382 2.3 0.00000937

*HOTAIR* 0.214 0.492 2.3 0.00000937

*TAC1* 0.186 0.427 2.29 0.00000937

*HOXB13* 0.147 0.334 2.28 0.00000937

*TBX20* 0.187 0.425 2.28 0.00000937

*PAX6* 0.18 0.408 2.27 0.00000937

*HOXB8* 0.162 0.367 2.27 0.00000937

*C14orf23* 0.178 0.402 2.26 0.00000937

*ONECUT1* 0.162 0.366 2.25 0.00000937

*HOXD12* 0.229 0.513 2.23 0.00000937

*PENK* 0.201 0.441 2.2 0.00000937

*ZNF454* 0.226 0.497 2.2 0.00000937

*LOC147804* 0.129 0.284 2.19 0.00000937

*LHX2* 0.212 0.464 2.19 0.00000937

*SOX1* 0.197 0.428 2.18 0.00000937

*PRDM13* 0.19 0.412 2.16 0.00000937

*PHYHIPL* 0.214 0.463 2.16 0.00000937

*PHOX2B* 0.201 0.436 2.16 0.00000937

*ZIC4* 0.234 0.505 2.16 0.00000937

*GRIA2* 0.224 0.479 2.14 0.00000937

*NBLA00301* 0.191 0.409 2.14 0.00000937

*HOXD10* 0.283 0.601 2.12 0.00000937

*GHSR* 0.224 0.474 2.12 0.00000937

*CSDAP1* 0.252 0.53 2.1 0.00000937

*ZNF833* 0.273 0.566 2.08 0.00000937

*TFAP2D* 0.163 0.337 2.07 0.00000937

*CDKN2A* 0.068 0.141 2.07 0.00000937

*LHX5* 0.155 0.32 2.06 0.00000937

*LHX8* 0.201 0.413 2.05 0.00000937

*SSTR1* 0.204 0.418 2.05 0.00000937

*T* 0.243 0.496 2.04 0.00000937

*NXPH1* 0.219 0.447 2.04 0.00000937

*HOXC12* 0.184 0.373 2.03 0.00000937

*CALCA* 0.195 0.394 2.02 0.00000937

*ZNF660* 0.168 0.34 2.02 0.00000937

*DRD5* 0.292 0.589 2.02 0.00000937

*PCSK1* 0.225 0.45 2 0.00000937

*BHLHE23* 0.065 0.44 6.82 0.0000187

*MIR129-2* 0.088 0.482 5.49 0.0000187

*MIR124-3* 0.089 0.474 5.33 0.0000187

*ZNF43* 0.084 0.412 4.91 0.0000187

*NKX6-2* 0.08 0.381 4.79 0.0000187

*DPY19L2* 0.083 0.379 4.57 0.0000187

*ZNF486* 0.086 0.373 4.33 0.0000187

*DOK5* 0.084 0.332 3.98 0.0000187

*ZNF582* 0.124 0.471 3.79 0.0000187

*FABP5L3* 0.092 0.343 3.71 0.0000187

*ZNF93* 0.054 0.198 3.64 0.0000187

*LOC100128811* 0.102 0.354 3.49 0.0000187

*CDX2* 0.109 0.359 3.29 0.0000187

*UTF1* 0.16 0.514 3.21 0.0000187

*MARCH11* 0.174 0.512 2.94 0.0000187

*HOXD9* 0.205 0.587 2.86 0.0000187

*HOXC9* 0.15 0.419 2.79 0.0000187

*MIR196A2* 0.191 0.531 2.78 0.0000187

*MYOD1* 0.115 0.314 2.73 0.0000187

*LOC401097* 0.066 0.169 2.56 0.0000187

*NPBWR1* 0.103 0.259 2.53 0.0000187

*ZNF880* 0.146 0.365 2.5 0.0000187

*LOC441177* 0.093 0.23 2.47 0.0000187

*C20orf103* 0.145 0.353 2.44 0.0000187

*ZNF781* 0.196 0.457 2.34 0.0000187

*CACNG8* 0.203 0.47 2.31 0.0000187

*SOX11* 0.234 0.528 2.26 0.0000187

*ZNF737* 0.151 0.339 2.24 0.0000187

*PRR15* 0.11 0.246 2.24 0.0000187

*GALR1* 0.15 0.328 2.18 0.0000187

*TRH* 0.246 0.534 2.17 0.0000187

*NEFH* 0.253 0.548 2.16 0.0000187

*CLK1* 0.046 0.099 2.13 0.0000187

*WT1* 0.224 0.473 2.12 0.0000187

*ZNF418* 0.278 0.579 2.08 0.0000187

*GPR120* 0.171 0.353 2.06 0.0000187

*ZNF626* 0.101 0.506 5 0.0000281

*ZNF702P* 0.096 0.397 4.15 0.0000281

*ZNF492* 0.104 0.395 3.78 0.0000281

*PTF1A* 0.124 0.448 3.61 0.0000281

*ZNF570* 0.11 0.364 3.31 0.0000281

*NEUROG3* 0.074 0.231 3.11 0.0000281

*RAB39* 0.061 0.171 2.82 0.0000281

*ACTA1* 0.163 0.424 2.61 0.0000281

*FGF4* 0.134 0.348 2.6 0.0000281

*SIX6* 0.184 0.472 2.56 0.0000281

*KHDRBS2* 0.171 0.424 2.48 0.0000281

*EVX2* 0.21 0.508 2.42 0.0000281

*LY6H* 0.156 0.33 2.12 0.0000281

*ZIC1* 0.265 0.551 2.08 0.0000281

*POU4F1* 0.11 0.433 3.92 0.0000468

*ZNF382* 0.114 0.441 3.85 0.0000468

*ZNF471* 0.106 0.409 3.85 0.0000468

*POU3F3* 0.144 0.545 3.79 0.0000468

*HMX3* 0.074 0.259 3.51 0.0000468

*MIR137* 0.139 0.438 3.15 0.0000468

*GPR149* 0.108 0.338 3.14 0.0000468

*UNCX* 0.128 0.397 3.1 0.0000468

*BARHL1* 0.137 0.371 2.72 0.0000468

*CXCL6* 0.152 0.399 2.63 0.0000468

*GLRA3* 0.099 0.255 2.59 0.0000468

*EPHA5* 0.141 0.363 2.57 0.0000468

*CCNA1* 0.183 0.42 2.29 0.0000468

*LOC157627* 0.163 0.37 2.28 0.0000468

*HMX2* 0.186 0.42 2.26 0.0000468

*FOXI2* 0.223 0.497 2.23 0.0000468

*HKR1* 0.284 0.603 2.12 0.0000468

*GJD2* 0.231 0.478 2.07 0.0000468

*SVIP* 0.041 0.427 10.32 0.0000656

*ZNF583* 0.104 0.445 4.27 0.0000656

*SLC2A10* 0.078 0.195 2.51 0.0000656

*HBM* 0.082 0.205 2.5 0.0000656

*ZNF577* 0.299 0.638 2.14 0.0000656

*DKK2* 0.211 0.427 2.02 0.0000656

*KAAG1* 0.255 0.513 2.01 0.0000656

*DBX1* 0.152 0.389 2.56 0.0000937

*GREB1L* 0.145 0.372 2.56 0.0000937

*TWIST1* 0.116 0.282 2.44 0.0000937

*CHAD* 0.25 0.597 2.39 0.0000937

*ZNF738* 0.178 0.375 2.11 0.0000937

*LRAT* 0.173 0.36 2.09 0.0000937

*WDR69* 0.249 0.513 2.06 0.0000937

*CDK5R2* 0.132 0.266 2.02 0.0000937

*NRIP2* 0.307 0.615 2 0.0000937

*LOC283392* 0.081 0.342 4.19 0.000131

*DPY19L2P4* 0.099 0.289 2.93 0.000131

*FOXB1* 0.122 0.343 2.81 0.000131

*NKX2-2* 0.114 0.292 2.56 0.000131

*HOXA9* 0.248 0.547 2.21 0.000131

*ZNF347* 0.18 0.386 2.15 0.000131

*LOC283731* 0.112 0.229 2.04 0.000131

*LOC100130148* 0.111 0.223 2 0.000131

*NKX2-3* 0.11 0.288 2.61 0.000178

*C3orf15* 0.096 0.227 2.36 0.000178

*OLFM3* 0.181 0.368 2.03 0.000178

*ZNF708* 0.075 0.149 2 0.000178

*OLIG1* 0.131 0.336 2.56 0.000234

*PTGFR* 0.142 0.329 2.32 0.000234

*ZNF569* 0.182 0.402 2.21 0.000234

*ZNF470* 0.169 0.373 2.2 0.000234

*RIPPLY2* 0.072 0.352 4.86 0.000309

*ZNF568* 0.094 0.341 3.62 0.000309

*MIR125B1* 0.229 0.663 2.9 0.000309

*ZNF85* 0.135 0.368 2.74 0.000309

*ZNF829* 0.15 0.375 2.49 0.000309

*GPR135* 0.195 0.453 2.32 0.000309

*ZNF804A* 0.159 0.34 2.13 0.000309

*FBLL1* 0.236 0.499 2.11 0.000309

*GABRA2* 0.092 0.34 3.68 0.000403

*FGF10* 0.124 0.325 2.62 0.000403

*ZNF790* 0.099 0.256 2.57 0.000403

*RAB6C* 0.135 0.333 2.47 0.000403

*TMEM229A* 0.189 0.405 2.14 0.000403

*DMRTA2* 0.194 0.412 2.12 0.000403

*GSC2* 0.092 0.244 2.66 0.000515

*SNAP91* 0.17 0.429 2.53 0.000515

*RERG* 0.116 0.319 2.76 0.000656

*SLCO4C1* 0.196 0.452 2.31 0.000656

*MIR935* 0.291 0.591 2.03 0.000656

*ZNF763* 0.087 0.241 2.76 0.00103

*ID4* 0.087 0.188 2.17 0.00103

*ZNF671* 0.249 0.543 2.18 0.00128

*C5orf38* 0.137 0.39 2.84 0.00194

*ZNF69* 0.164 0.332 2.02 0.00194

*LXN* 0.194 0.517 2.67 0.00288

*STK33* 0.239 0.516 2.16 0.00288

*ISL2* 0.184 0.374 2.03 0.00288

*C21orf88* 0.08 0.234 2.93 0.00347

*VENTX* 0.138 0.279 2.02 0.00347

*EID3* 0.178 0.526 2.96 0.00419

*MIR1253* 0.084 0.246 2.92 0.00419

*TFPI2* 0.147 0.359 2.44 0.00419

*QRFPR* 0.212 0.473 2.23 0.00419

*FOXA1* 0.08 0.188 2.35 0.00502

*ZFP28* 0.157 0.422 2.69 0.00599

*MMP23B* 0.164 0.401 2.45 0.00599

*EPHA7* 0.168 0.398 2.37 0.00599

*SLC46A3* 0.209 0.476 2.27 0.00847

*HELT* 0.073 0.155 2.14 0.01

*RNF180* 0.111 0.223 2 0.01

*ZNF214* 0.175 0.355 2.03 0.0222

*ZNF323* 0.194 0.468 2.42 0.0259

*ASXL3* 0.042 0.087 2.08 0.0259

*C17orf46* 0.231 0.533 2.31 0.0349

*LOC389705* 0.144 0.354 2.47 0.0403

*GPR150* 0.148 0.348 2.35 0.0465

## Supplementary Table 3: Lack of link between *MEOX2*, *CBLN1*, *OLIG2* and *SOX14* methylation levels and clinical parameters (age, sex, smoking history, alcohol use and T and N categories) in TCGA-OSCC.

***CBLN1* *MEOX2* *OLIG2* *SOX14***

**Age** 0.9093 0.9723 1 0.9491

**Sex** 1 0.9132 0.9132 0.6780

**Smoking**  0.7564 1 0.9132 1

**Alcohol** 1 1 0.6780 0.9132

**T** 0.9723 1 1 1

**N** 0.7852 0.6780 0.6780 0.6780

## Showing *p* values using Student’s t-test or Chi2, p<0.05 used as threshold for significance (with FDR correction). Tumors are stratified according to the median value of DNA methylation levels for each gene to define groups with low or high DNA methylation.

## Supplementary Table 4: ROC analyses for individual genes and gene combinations

**Methylation status AUC [CI95%] *p* value**

*ASCL1* 0.55 [0.29 – 0.80] 0.3655

*MEOX2* 0.66 [0.45 – 0.87] 0.0692

*CBLN1* 0.57 [0.43 – 0.71] 0.1587

*OLIG2* 0.50 [0.50 – 0.50] NA

*SOX14* 0.60 [0.40 – 0.80] 0.1587

*ASCL1+MEOX2* 0.63 [0.39 – 0.88] 0.1473

*MEOX2+CBLN1* 0.72 [0.50 – 0.93] 0.0229 *

*MEOX2+SOX14* 0.67 [0.46 – 0.88] 0.0587

*MEOX2+CBLN1+SOX14* 0.72 [0.51 – 0.93] 0.0180 *

*ASCL1+MEOX2+CBLN1+SOX14* 0.71 [0.47 – 0.94] 0.0429 *
